# From the local disparities to national realities: Mapping and multilevel modeling of catastrophic health expenditure in Bangladesh using HIES 2016

**DOI:** 10.1371/journal.pone.0290746

**Published:** 2024-01-02

**Authors:** Md. Muhitul Alam, Md. Israt Rayhan, Mohaimen Mansur

**Affiliations:** Institute of Statistical Research and Training (ISRT), University of Dhaka, Dhaka, Bangladesh; Federal Teaching Hospital, Ido-Ekiti, NIGERIA

## Abstract

In developing nations, catastrophic health expenditures have become an all-too-common occurrence, threatening to push households into impoverishment and poverty. By analyzing the Household Income and Expenditure Survey 2016, which features a sample of 46,080 households, this study provides a comprehensive district-by-district analysis of the variation in household catastrophic health expenditures and related factors. The study utilizes a multilevel logistic regression model, which considers both fixed and random effects to identify factors associated with catastrophic health expenditure. The findings of the study indicate that districts located in the eastern and southern regions are at a significantly higher risk of experiencing catastrophic health expenditures. A potential explanation for this trend may be attributed to the high prevalence of chronic diseases in these districts, as well as their economic conditions. The presence of chronic diseases (AOR 5.45 with 95% CI: 5.14, 5.77), presence of old age person (AOR 1.50 with 95% CI: 1.39, 1.61), place of residence (AOR 1.40 with 95% CI: 1.14, 1.73) are found to be highly associated factors. Additionally, the study reveals that the thresholds used to define catastrophic health expenditures exhibit substantial variation across different regions, and differ remarkably from the threshold established by the WHO. On average, the thresholds are 23.12% of nonfood expenditure and 12.14% of total expenditure. In light of these findings, this study offers important insights for policymakers and stakeholders working towards achieving universal health coverage and sustainable development goals in Bangladesh.

## Introduction

Accessing health care services is essential for individuals to maintain their health and well-being. However, in many low- and middle-income countries (LMICs), the exorbitant costs associated with these services have become a recurring problem that eventually forces households into poverty [1]. Out-of-pocket (OOP) health payments, which refer to the expenditures made by families at the time they obtain health services, often include doctor’s consultation fees, medicine purchases, and hospital expenses that are not covered by insurance [2]. Catastrophic health expenditure (CHE) is incurred when a family’s OOP costs exceed a certain threshold, and this can have severe financial and social consequences for families in LMICs [3, 4].

The issue of OOP expenditure is particularly concerning in Bangladesh, where it is a significant barrier to achieving universal health coverage (UHC), a key target of the Sustainable Development Goals (SDGs) [5]. The repercussions of excessive OOP spending are immense, with many households being forced to sell their assets, ration their food, and cut back on essential expenses which eventually lead to malnutrition [6]. Children’s education may also be negatively impacted by out-of-pocket medical expenses, causing a cycle of poverty and deprivation [7]. In addition, out-of-pocket payments may cause planners and policymakers to miscalculate poverty status [5]. Each year, approximately 150 million individuals face financial catastrophe, and 100 million are driven below the poverty line because they must pay for health services. More than 90% of these individuals reside in low-income nations [8]. Although only 2.4% of Bangladesh’s GDP was allocated to healthcare in 2019, 63% of all healthcare costs are covered by OOP payments [9]. Bangladesh has the second-highest proportion (5.1%) of total household resources devoted to out-of-pocket medical expenses among 11 low- to middle-income Asian nations trailing only Vietnam. Bangladesh’s poverty rate climbed by almost four percent (equivalent to nearly 5 million more people considered poor) at the $1.08 poverty line when out-of-pocket expenses were subtracted from total resources [10].

In Bangladesh, Rahman et al., 2020 found that paralysis, liver disorders, injuries, and heart diseases incurred significant expenses and were associated with a high prevalence of CHE [11]. Similarly, Sheikh et al., 2022 reported that non-communicable diseases have higher OOP costs than communicable diseases, with cancer incurring the highest OOP cost, followed by liver disease and heart illness [12]. Regions with a high incidence of disease, such as river islands and water-submerged areas, have higher average OOP expenses [13, 14]. In terms of spending, medicine costs account for approximately 60% of direct OOP expenditures [15–17]. The presence of chronic disease increases healthcare costs [18]. Older, less educated, never-married, and non-working household heads are more likely to experience CHE due to medication expenses [19]. A study conducted in Poland found a causal and positive relationship between CHE and relative poverty [20]. In Bangladesh, 3.2% of households are not considered poor but become impoverished due to OOP expenses, resulting in an 8.8% undercount of poverty [21–23].

According to the WHO’s definition, catastrophic health expenditures arise when a family’s health-related expenses account for at least 40% of its non-food expenditures while World Bank suggests 10% of total expenditure as the threshold [24]. It was also discovered that the threshold differed from one nation to another and from one wealth class to a different one [25, 26]. Studies conducted in Bangladesh have also employed a variety of cutoffs. Ahmed et al., 2022, Rahman et al., 2013, Xu et al., 2003 employed a threshold of 10% of total expenditure and 40% of nonfood expenditure [13, 27, 28], whereas Molla and Chi, 2018 utilized a range of cutoffs to determine how the outcomes varied [22]. Instead of the 40% cutoff point of nonfood expenditure, Khan et al. (2017) set it at 25% [29].

The main objectives of this research are twofold: firstly, to identify the districts in Bangladesh that are most vulnerable to experiencing catastrophic health expenditure (CHE); and secondly, to investigate whether the threshold for defining CHE varies across regions and how it compares to other established thresholds. The novelty of this study is to identify the administrative areas (districts) in Bangladesh that are most susceptible to incurring catastrophic health care costs.

**Fig 1:**
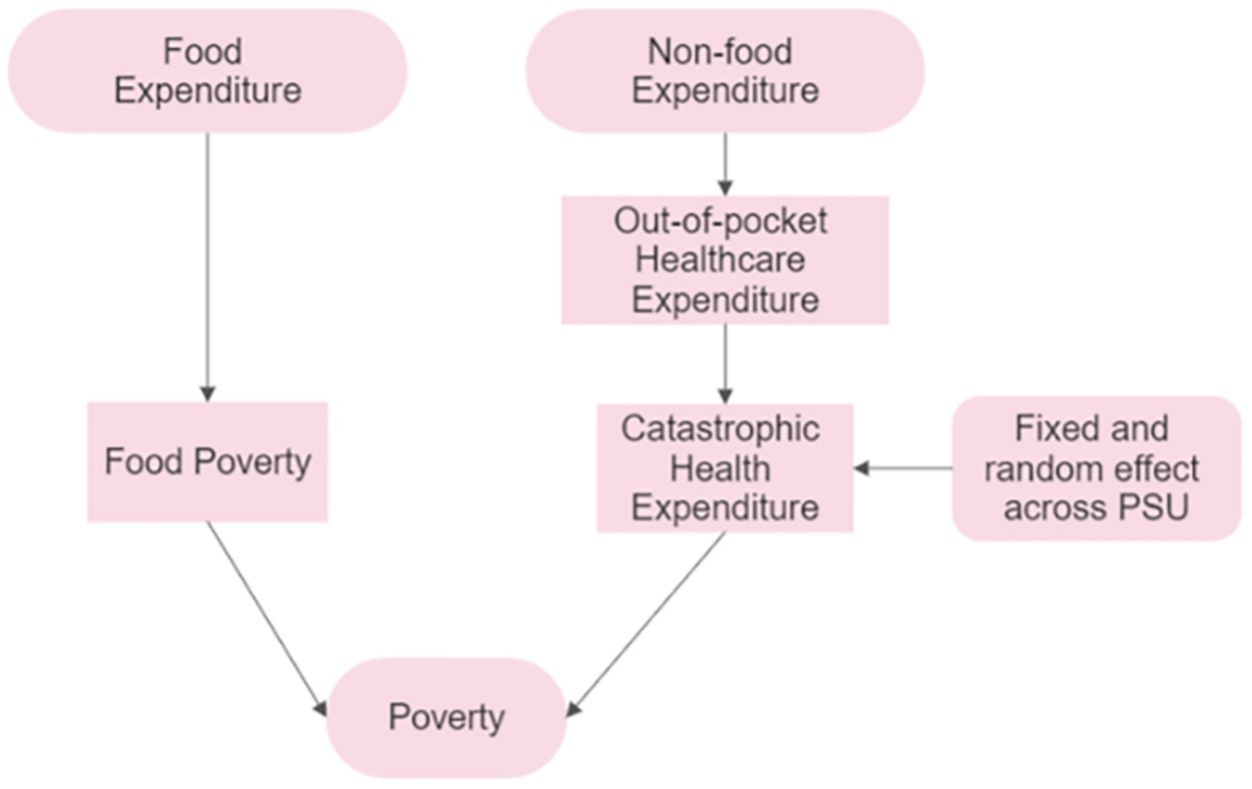
Conceptual framework (Source: Author’s own compilation).

A nationally representative dataset is used to capture the hierarchical nature and to identify factors associated with CHE. Fig 1 visually represents the conceptual framework of this study. The findings of this research may figure out the region-specific intervention strategies and policies that can contribute to achieving universal health coverage, a key Sustainable Development Goal.

## Methodology

### Data source and variables

The study is based on the existing secondary data from the Household Income and Expenditure Survey (HIES) 2016, conducted by the Bangladesh Bureau of Statistics (BBS) every five years. The survey was carried out during April 1, 2016—March 31, 2017 and employed a two-stage stratified cluster sampling method, where 36 primary sampling units (PSUs) were chosen from each district using the probability proportional to size (PPS) systematic sampling technique. The PSUs were selected from the enumeration areas (EAs) of the Bangladesh Census of Population and Housing, with each EA representing an average of 110 households. In the second stage, 20 households were randomly selected from each PSU, resulting in a total of 46,080 households being selected for the survey, of which 46,076 participated [30].

This study does not contain any clinical studies or involve any human participants performed by any of the authors. An oral consent was taken from each of the respondents before the interview. All identification of the respondents was unidentified before publishing data. The secondary data sets analyzed in the current study are freely available upon registration and request from the BBS website [30]. In this study, the response variable examined is catastrophic health expenditure (CHE), a binary variable. Two unique definitions of CHE were applied here. The first definition states that CHE has been incurred in a household when its monthly health expenditures exceed 40% of its nonfood expenditures. The second definition states that CHE has been incurred in a household when its monthly health expenditures exceed 10% of its total expenditures. Considering the fact that there exists a causal relation between CHE and poverty and several literatures such as Wagstaff and Doorslaer, 2003 defined CHE as the amount of health expenditure that drives households below the poverty line, a region-specific threshold for CHE is constructed below [31]:

Let, $y_{i}^{t}$, $y_{i}^{nf}$, and $y_{i}^{h}$ denote the per capita consumption expenditure, per capita nonfood expenditure and per capita health expenditure of the *i*th household, respectively. Let, *z* denote the per capita average poverty line. Suppose, *n*_*j*_ is the number of households in the *j*th region where *j* = 1,2, …,16. Consider the poverty indicator *P*_*i*_ such that,

$$P_{i}=\left\{\begin{array}{ll} 1, & y_{i}^{t}<z\\ 0, & otherwise. \end{array}\right.$$

Then, the following steps are followed to calculate the data driven region-specific thresholds:

Step 1: $y_{i}^{t^{\prime }}=y_{i}^{t}-y_{i}^{h}$
Step 2: Calculate $P_{i}^{\prime }$ where,

$$P_{i}^{\prime }=\left\{\begin{array}{ll} 1, & y_{i}^{t^{\prime }}<z\\ 0, & otherwise \end{array}\right.$$

Step 3: Identify the households with *P*_*i*_ = 0 but $P_{i}^{\prime }=1$.
Step 4: For each region, calculate: ${\text{t}}_{\text{j}}=\frac{\text{1}}{{\text{n}}_{\text{k}}}\sum_{\text{k}=\text{1}}^{{\text{n}}_{\text{k}}}\frac{{\text{y}}_{\text{k}}^{\text{h}}}{{\text{y}}_{\text{k}}^{\text{t}}}$, for the households satisfying *P*_*i*_ = 0 but $P_{i}^{\prime }=1$. Here, $\frac{{\text{y}}_{\text{k}}^{\text{h}}}{{\text{y}}_{\text{k}}^{\text{t}}}$ denotes the proportion of total expenditure spent on health by the *k*th household in *j*th region. Then, *t*_*j*_ is called the data driven region specific threshold for CHE for region *j*. In brief, the threshold for each region represents the percentage of total household expenses allocated to healthcare by households in that region who were not classified as poor initially but became poor after deducting healthcare expenses from their total expenditure. Similarly, to calculate the data driven region-specific nonfood expenditure threshold, simply replace $y_{k}^{t}$ with $y_{k}^{nf}$ in step 4.

The poverty line calculated by BBS in their HIES 2016 is used here. BBS employed the Cost of Basic Needs (CBN) method to calculate the poverty line. The detailed method of calculating the poverty line can be found in the final report of HIES 2016 [30]. The independent variables of the study were chosen based on literature. These variables include: Presence of persons over 65 years in the household (No, Yes), Wealth index (Poorest, Second, Third, Fourth, Richest), Presence of chronic disease (No, Yes), Household head received education (No, Yes), Sex of household head (Female, Male), Age of household head (Less than 60 years, Over 60 years), Division (Barisal, Chittagong, Dhaka, Khulna, Mymensingh, Rajshahi, Rangpur, Sylhet), Place of residence (Rural, Urban, City corporation). The variable wealth index was constructed through principal component analysis (PCA). First, variables required to construct wealth index were chosen based on the suggestion of the Demographic and Health Survey (DHS) program [32]. Then, after applying PCA, the scores of first principal component were extracted. These scores were divided into 5 groups from which the wealth indexes of the households are derived.

### Methods

To explore the possibility of an association between the covariates and the dependent variable, a bivariate analysis and Pearson’s chi-square test are carried out. The chi-square test is a nonparametric statistical method for determining whether or not two categorical variables are significantly related to one another [33]. Although bivariate analysis can provide a basic understanding of the association between a dependent and independent variable, these results are not adjusted for potential confounding variables. The statistical model known as binary logistic regression is used to measure the association between a binary dependent variable (a variable that can only take on one of two potential values) and one or more independent variables.

If we consider multiple explanatory variables, then the logistic model is called a multiple logistic regression model. Consider **X** = (*X*_1_, *X*_2_, …, *X*_*p*_) as the set of explanatory variables in the model and **x** = (*x*_1_, *x*_2_, …, *x*_*p*_) is their observed values. Then the multiple logistic regression model is given by:

$$logit(\pi (x))=log\frac{\pi (x)}{1-\pi (x)}=\beta_{0}+\beta_{0}x_{1}+\beta_{2}x_{2}+\cdots +\beta_{p}x_{p},$$

where *β*_*i*_’s are the parameters of the model for *i = 1*,*2*, *…*, *p*.

A multilevel logistic regression is a model used to analyze a data with hierarchical structure where the response variable is a binary variable. In contrast to the traditional logistic regression, multilevel logistic regression allows the log odds of the response variable being 1 rather than 0 to vary from one cluster to another. In a multilevel logistic regression model, both the slope and intercept can be assumed to be fixed or random across clusters. To choose the appropriate multilevel logistic regression model a three-step procedure proposed by Sommet and Morselli, 2017 can be followed [34].

Let, *Y*_*ij*_ denote the response of the *i*th individual in *j*th cluster and **X**_**ij**_ = (*X*_1*ij*_, *X*_2*ij*_, …, *X*_*pij*_) denote a set of explanatory variables for that individual and **x**_**ij**_ = (*x*_1*ij*_, *x*_2*ij*_, …, *x*_*pij*_) are its observed values. Let, *π*_*ij*_ denote the conditional probability that the response variable is 1 for *i*th individual in *j*th cluster.

Grand mean centering or cluster-mean centering is done on the lower-level predictors depending on whether general or cluster specific effects are desired. In the first step, we try to obtain how much the log odds of the response variable being 1 instead of zero vary from cluster to cluster. So, if a random intercept model is considered, it has the following form:

$$logit(\pi_{ij})=\text{log}\frac{\pi_{ij}}{1-\pi_{ij}}=\beta_{oo}+u_{oj}$$

where *β*_00_ is the fixed intercept and *u*_0*j*_ is the difference between fixed intercept and the intercept for the *j*th cluster. Then the Intraclass Correlation Coefficient (ICC) is calculated, which is given by:

$$ICC=\frac{var(u_{oj})}{var\left(u_{0j}\right)+\pi^{2}/3},\;$$

where *π*^2^/3 is the variance of the standard logistic distribution. So, Using the ICC, one may calculate the proportion of the total variance in *Y* that can be ascribed to differences between clusters. The value of the interclass correlation (ICC) can serve as a criterion for evaluating the necessity of employing multilevel modeling. Higher values suggest a stronger need for multilevel modeling, while lower values indicate that it may not be necessary [35]. Similar to the intercept, the slope of a predictor can likewise change from one cluster to another. A likelihood ratio test can be used to compare two models and determine whether the slope of a predictor should be kept random or fixed. The last step is to run the final model with desired fixed or random intercept and slope parameters.

A random intercept multilevel multiple logistic regression model is given by:

$$logit(\pi_{ij})=\text{log(}\frac{\pi_{ij}}{1-\pi_{ij}})=(\beta_{00}+u_{0j})+\beta_{01}x_{1ij}+\beta_{02}x_{2ij}+\cdots +\beta_{0p}x_{pij},$$

where *β*_0*j*_’s are the parameters of the multilevel multiple logistic regression model for *j = 1*,*2*, *…*, *p*.

Academic research frequently employs the receiver operating characteristic (ROC) curve to evaluate the performance of binary classifiers [36]. At various threshold levels, ROC curves depict the true positive rate (sensitivity) versus the false positive rate (1-specificity). The area under the ROC curve (AUC) is a commonly employed metric for measuring the overall effectiveness of a classifier, with an AUC of 1 representing a perfect classifier and an AUC of 0.5 representing a random guess [37]. ROC curves provide a graphical representation of the trade-off between sensitivity and specificity, and are especially useful when the cost of false positives and false negatives differ. Overall, ROC curves are a good way of assessing classifier performance and comparing various classification algorithms.

## Analysis and results

**Fig 2:**
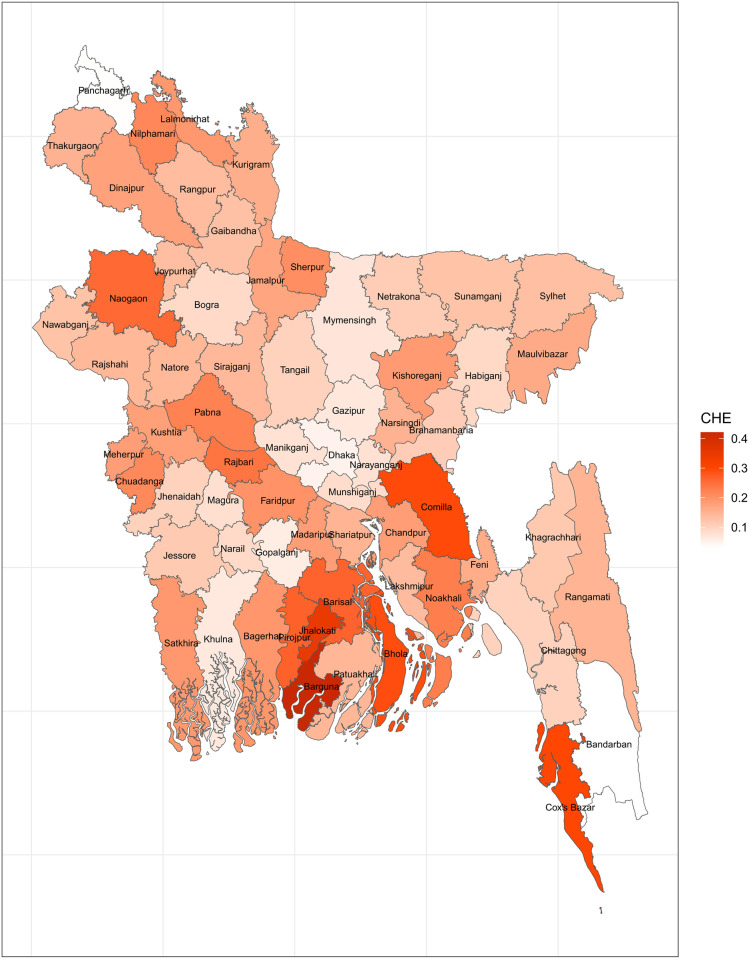
Mapping CHE using WHO 40% nonfood expenditure threshold (Source: Author’s own compilation).

A household’s health expenditures are deemed catastrophic when they reach 40% of its non-food expenditures [38]. This threshold has been observed in a number of published works such as Ahmed et al., 2022; Rahman et al., 2013; Xu et al., 2003. Considering that this threshold remains uniform across districts, Fig 2 offers a map of so-defined CHE. Apart from Comilla, Cox’s bazar, Barguna, Jhalokati, Bhola, Barisal, and a few other districts in the north of the nation, the prevalence of CHE is quite low across all of Bangladesh’s districts. Thus, when a threshold of 40% of non-food expenditures is applied, the prevalence of CHE appears to be rather tolerable.

**Fig 3:**
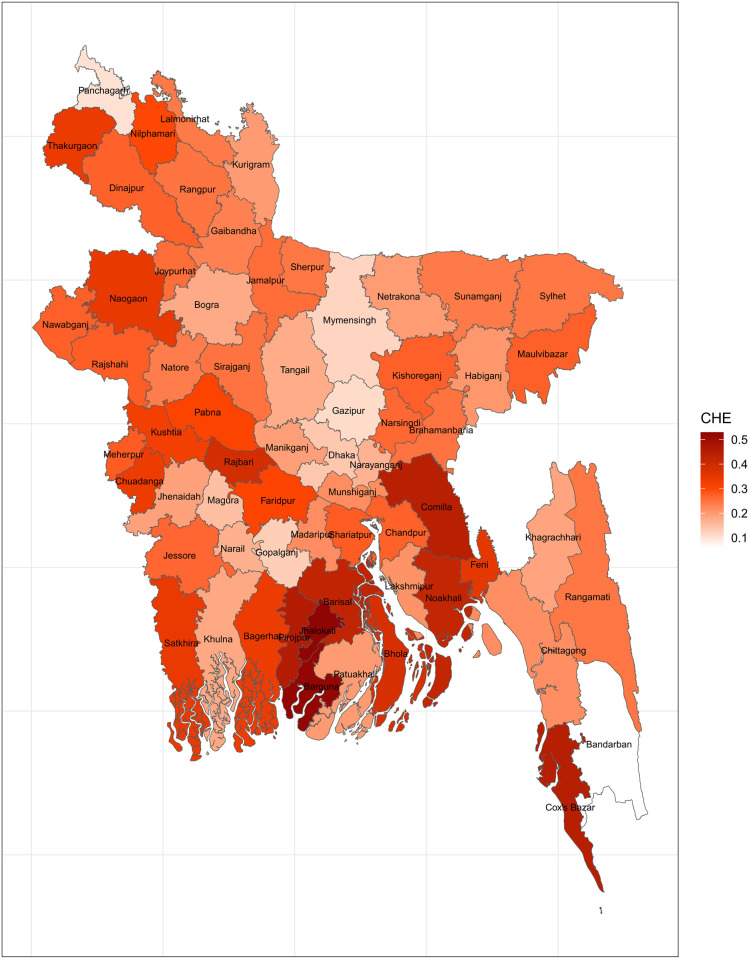
Mapping CHE using 10% total expenditure threshold (Source: Author’s own compilation).

Another definition of catastrophic health expenditure refers to household’s healthcare costs exceeding 10% of its total expenditures [38]. Assuming that this criterion is consistent across districts, a map of CHE is displayed in Fig 3. This map suggests that the prevalence of CHE is significantly higher over the entire nation. Comilla, Jhalokati, Barguna, Noakhali, Barisal, Bhola, and Cox’s bazaar have the highest prevalence of CHE. In addition to these districts, several others, including Rajbari, Thakurgaon, and Pirojpur, currently have moderately high CHEs. When this cutoff is used to define CHE, it appears to be a much larger problem.

One of the goals of this study is to examine if the cutoffs used to define CHE vary from one region to another. For this reason, we attempt to compute a region-specific, data-driven threshold. The thresholds are computed as average of OOP health expenditure as a percentage of nonfood (or total) expenditure for each region, where averaging is done over individuals who fall below the average poverty line after excluding health expenses. Table 1 reports the derived thresholds for each region.

**Table 1:** Data driven region-specific thresholds for CHE.

Region	Threshold (%nonfood)	Threshold (%total)
Barisal (Rural)	30.10	16.76
Barisal (Urban)	27.13	14.83
Chittagong (Rural)	20.38	10.18
Chittagong (Urban)	27.29	15.88
Chittagong (City Corp.)	23.55	13.41
Dhaka (Rural)	22.09	11.07
Dhaka (Urban)	21.69	11.26
Dhaka (City Corp.)	22.51	10.95
Khulna (Rural)	25.66	13.36
Khulna (Urban)	24.66	13.29
Khulna (City Corp.)	17.18	9.30
Rajshahi (Rural)	23.91	12.58
Rajshahi (Urban)	24.19	12.46
Rajshahi (City Corp.)	15.48	8.31
Sylhet (Rural)	20.39	8.96
Sylhet (Urban)	23.78	11.58

According to Table 1, the thresholds for nonfood expenditure and total expenditure vary considerably. The threshold is highest in Barisal (rural) and lowest in Rajhshahi (City Corporation) for both definitions. Although the region-specific threshold for total expenditure is relatively close to the previously defined and often used level of 10%, the region-specific threshold for nonfood expenditure is well below the WHO-specified threshold of 40%. The data-driven average regional thresholds are found to be 23.124% of nonfood expenditure and 12.137% of total expenditure.

**Fig 4:**
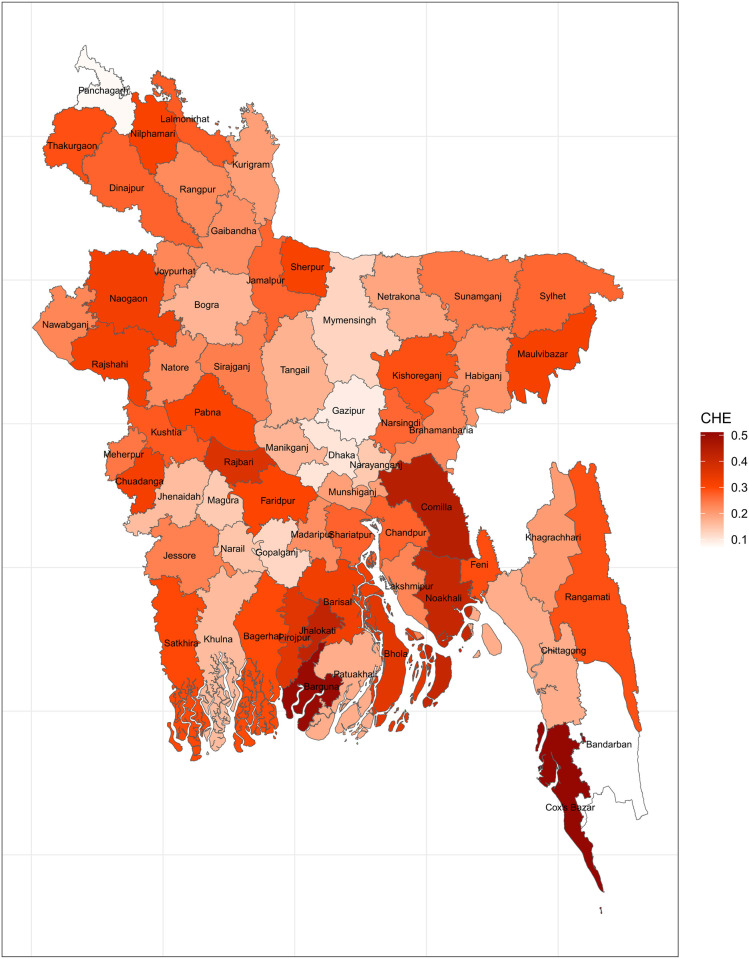
Mapping CHE using data-driven region-specific nonfood expenditure threshold (Source: Author’s own compilation).

Fig 4 illustrates the mapping of CHE using the region-specific threshold based on the household’s nonfood expenditures as the cutoff. This map displays results comparable to those achieved when 10% of total expenditures was chosen as the criterion. Jhalokati, Barguna, Noakhali, Barisal, Bhola, and Cox’s Bazar have the highest prevalence rates of CHE, although several other districts have somewhat high prevalence of CHE.

**Fig 5:**
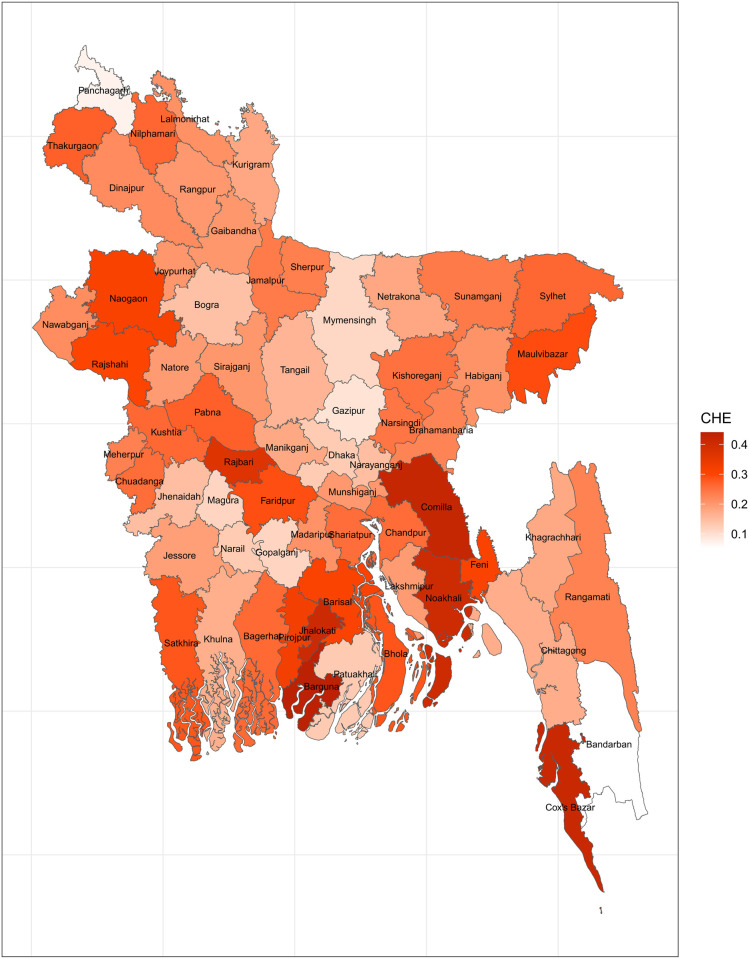
Mapping CHE using data-driven region-specific total expenditure threshold (Source: Author’s own compilation).

Fig 5 displays the mapping of CHE using the region-specific threshold based on the total household expenditures as the cutoff. Since, the region-specific thresholds based on total expenditure are not very different from the 10% threshold previously chosen, so the maps (Figs 3 and 5) are fairly similar.

**Fig 6:**
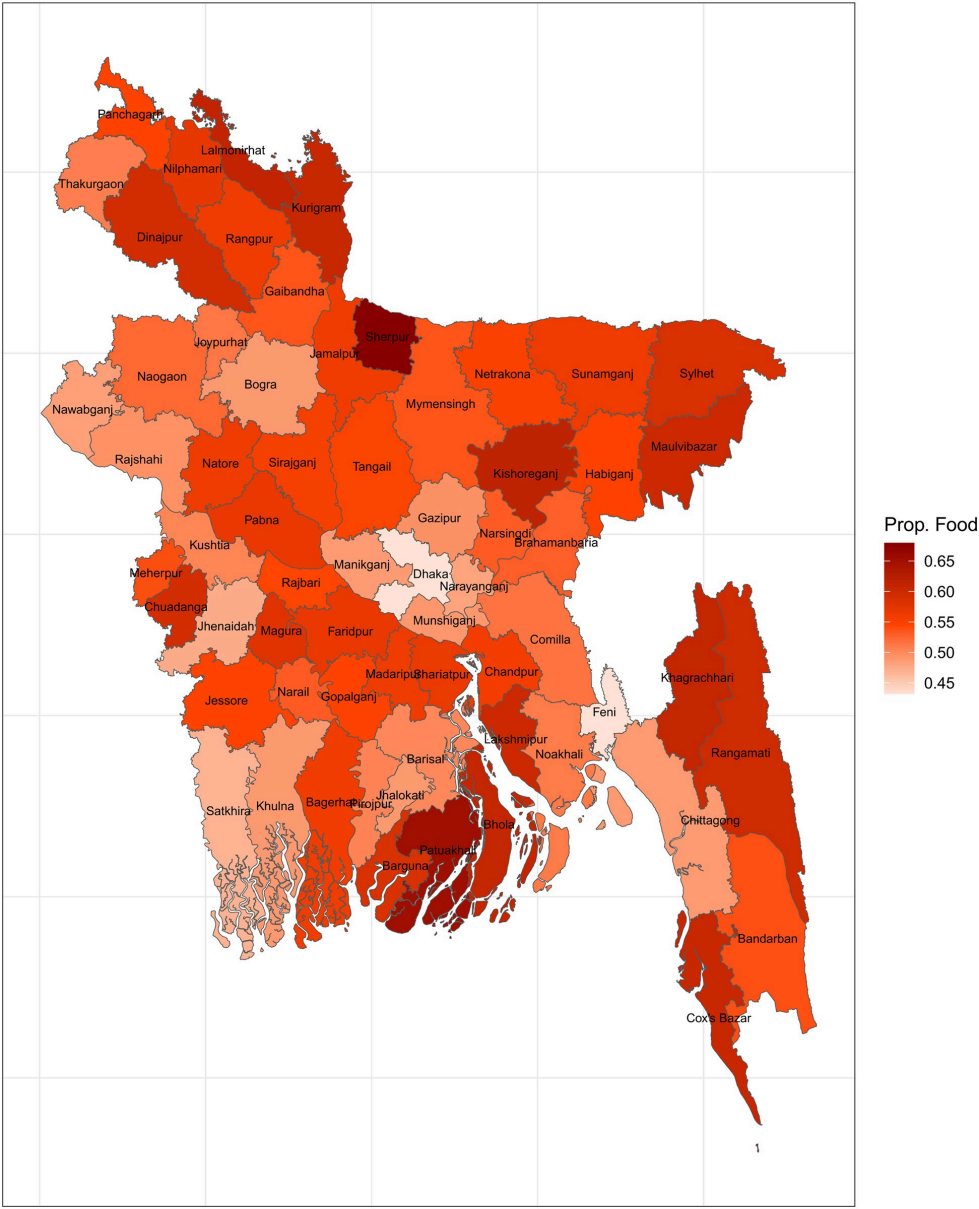
Proportion of food expenditure over the total expenditure (Source: Author’s own compilation).

The map of proportion of food expenditure slightly resembles previously constructed maps of CHE. Sherpur, Potuakhali, Cox’s bazaar, Bhola, and Barguna and some districts in the north have a high proportion of total expenditures devoted to food, as depicted in Fig 6. It is well-established that a greater share of a household’s expenditures on food suggests that the household belongs to a lower socioeconomic class. The slight similarity between Fig 6 and the CHE maps may be attributable to the association between CHE and the wealth index.

**Fig 7:**
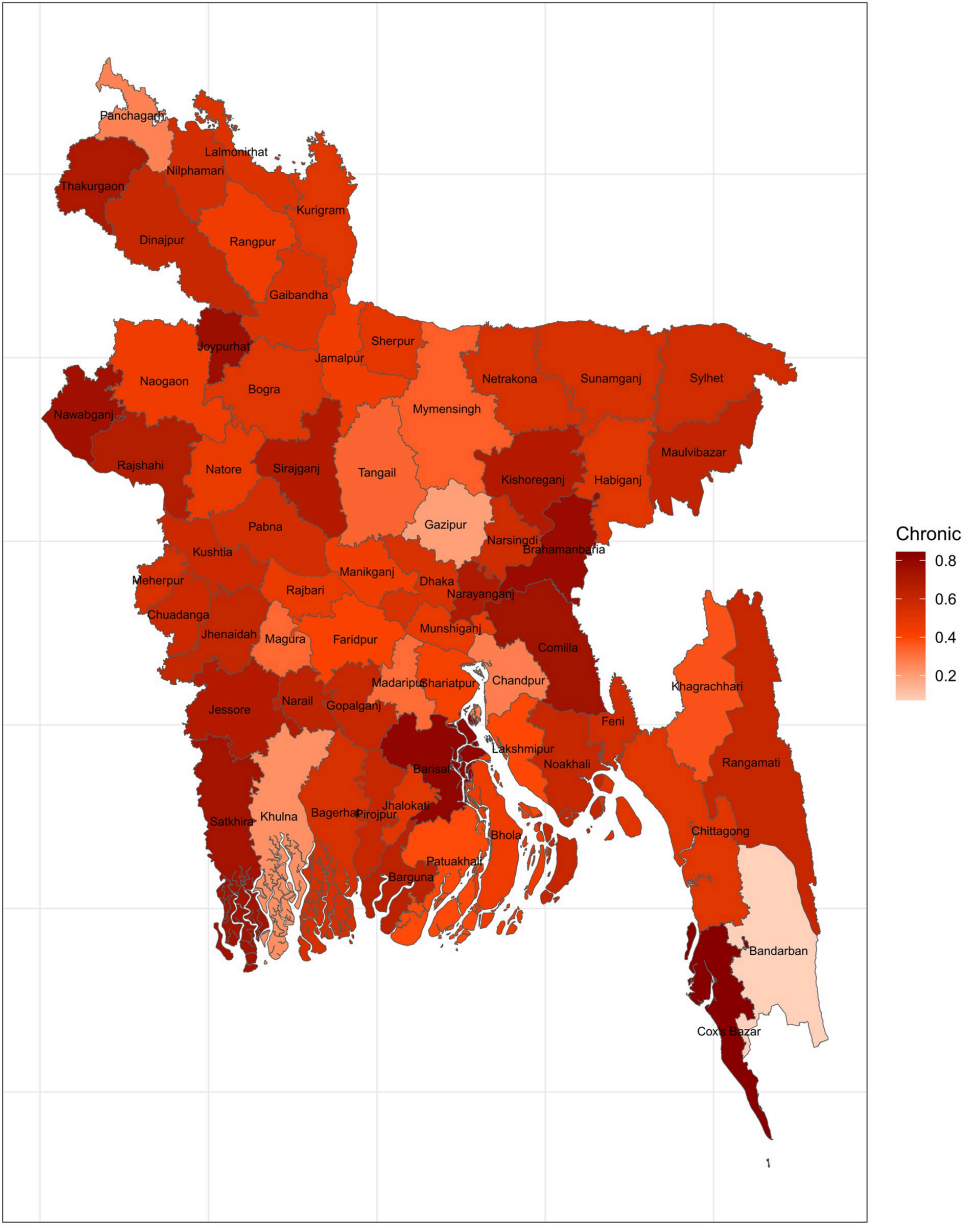
Prevalence of chronic disease across the districts (Source: Author’s own compilation).

Fig 7 shows how the prevalence of chronic disease varies across districts. Careful inspection of this figure reveals its similarities with the CHE maps. The prevalence of chronic disease was seen to be high in the districts Cox’s bazar, Barisal and Comilla. It should be noted that, these districts had very high prevalence of CHE as well.

The maps that display catastrophic health expenditure (CHE) demonstrate significant variations from the poverty map provided by HIES [30]. However, the map illustrating the proportion of food expenditure appears to closely resemble the poverty map. This indicates that CHE may not be the only factor contributing to the impoverishment of households in Bangladesh. These maps offer an initial insight into the relationship between CHE and other explanatory variables, indicating a possible association between CHE, chronic disease prevalence, and the wealth index. Regression model is used to investigate this assumption, and the resulting findings are adjusted for potential confounding variables.

The bivariate Table 2 depicts the distribution of explanatory variables within each outcome variable category. In this table, both nonfood and total expenditure-based data-driven region-specific thresholds are displayed. Pearson’s chi-square test is also used to examine the association between two variables. According to both definitions of CHE, the prevalence of CHE is found to be greater in households with elderly members. When the nonfood threshold is applied, the prevalence of CHE among households with at least one elderly person is 34%, but it drops to 22% when households without an elderly person are considered. When a threshold for total expenditures is applied, a similar situation occurs. Surprisingly, the prevalence of CHE is lower in households with at least one child younger than 15 years of age. As expected, the prevalence of CHE decreases as the household’s socioeconomic status rises. It is believed that chronic disease is strongly associated with CHE. 36% of households where at least one member had a chronic disease in the past year experienced CHE, compared to 10% of households where no member had a chronic disease in the past year. The relationship between the education level of the household head and CHE is significant when the nonfood threshold is used, but it is not significant when the total expenditure threshold is applied. The prevalence of CHE is higher in rural regions and lower in city corporations. Barisal has the highest prevalence of CHE among the administrative divisions. When the household head is female, the prevalence of CHE is slightly higher than that of the male, and when the household head is over 60 years old, it is associated with a higher prevalence of CHE.

**Table 2:** Bivariate summary table showing the distribution of all the predictors across the categories of CHE.

Variables	CHE (data-driven nonfood)		CHE (data-driven total)	
	Yes, N = 10,879	p-value	Yes, N = 12,051	p-value
**Presence of old age (65+)**		<0.001		<0.001
No	22.0%		24.4%	
Yes	34.4%		37.4%	
**Presence of child (< 15)**		<0.001		<0.001
No	26.5%		29.3%	
Yes	22.8%		25.2%	
**Wealth Index**		<0.001		<0.001
Poorest	25.9%		25.9%	
Second	23.7%		24.7%	
Third	25.0%		27.1%	
Fourth	23.9%		27.1%	
Richest	20.5%		27.1%	
**Presence of chronic disease**		<0.001		<0.001
No	10.0%		11.6%	
Yes	36.2%		39.6%	
**Head received education**		<0.001		0.8
No	25.5%		26.4%	
Yes	22.6%		26.3%	
**Place of residence**		<0.001		<0.001
City Corporation	14.6%		19.9%	
Rural	25.5%		27.4%	
Urban	20.9%		24.6%	
**Division**		<0.001		<0.001
Barisal	39.2%		42.0%	
Chittagong	25.1%		28.2%	
Dhaka	18.9%		22.0%	
Khulna	22.7%		25.7%	
Mymensingh	20.3%		20.5%	
Rajshahi	23.5%		26.6%	
Rangpur	22.4%		24.1%	
Sylhet	22.8%		24.1%	
**Sex of household head**		0.6		0.014
Female	24.1%		27.7%	
Male	23.8%		26.2%	
**Age of household head**		<0.001		<0.001
60+	33.8%		36.6%	
Under 60 years	22.1%		24.6%	

Table 3 displays the results of a logistic and a multilevel logistic regression model in which the response variable catastrophic health expenditure takes the value 1 when a household’s health expenditures exceed its nonfood expenditure data-driven region-specific thresholds and takes the value 0 otherwise. This criterion is chosen because it accurately reflects the impoverishment impact of CHE and is widely utilized in the literature. Considering the structure of the survey design, the PSU’s are chosen as level 2 of the multilevel model and households are considered level 1.

**Table 3:** Logistic and multilevel logistic regression model results with nonfood expenditure data-driven region-specific threshold.

Variables	Logistic			Multilevel Logistic		
	Adj. OR	95% CI	p-value	Adj. OR	95% CI	p-value
**Presence of old age person (65+)**						
No	1.00	—		1.00	—	
Yes	1.48	(1.38, 1.58)	**<0.001**	1.50	(1.39, 1.61)	**<0.001**
**Presence of child (< 15)**						
No	1.00	—		1.00	—	
Yes	0.74	(0.70, 0.78)	**<0.001**	0.72	(0.68, 0.77)	**<0.001**
**Wealth Index**						
Poorest	1.00	—		1.00	—	
Second	0.88	(0.81, 0.94)	**<0.001**	0.86	(0.80, 0.93)	**<0.001**
Third	0.89	(0.83, 0.96)	<**0.002**	0.87	(0.80, 0.94)	**<0.001**
Fourth	0.82	(0.76, 0.88)	**<0.001**	0.80	(0.74, 0.87)	**<0.001**
Richest	0.67	(0.62, 0.73)	**<0.001**	0.66	(0.61, 0.72)	**<0.001**
**Presence of chronic disease**						
No	1.00	—		1.00	—	
Yes	5.52	(5.24, 5.83)	**<0.001**	5.45	(5.14, 5.77)	**<0.001**
**Place of residence**						
City Corporation	1.00	—		1.00	—	
Rural	1.62	(1.42, 1.85)	**<0.001**	1.64	(1.34, 2.01)	**<0.001**
Urban	1.40	(1.22, 1.61)	**<0.001**	1.40	(1.14, 1.73)	**<0.002**
**Division**						
Barisal	1.00	—		1.00	—	
Chittagong	0.54	(0.49, 0.59)	**<0.001**	0.51	(0.44, 0.59)	**<0.001**
Dhaka	0.39	(0.35, 0.42)	**<0.001**	0.37	(0.32, 0.43)	**<0.001**
Khulna	0.44	(0.40, 0.48)	**<0.001**	0.43	(0.37, 0.50)	**<0.001**
Mymensingh	0.39	(0.35, 0.44)	**<0.001**	0.37	(0.30, 0.45)	**<0.001**
Rajshahi	0.40	(0.36, 0.44)	**<0.001**	0.39	(0.33, 0.46)	**<0.001**
Rangpur	0.42	(0.38, 0.46)	**<0.001**	0.40	(0.34, 0.47)	**<0.001**
Sylhet	0.42	(0.37, 0.47)	**<0.001**	0.40	(0.33, 0.49)	**<0.001**
**Head received education**						
No	1.00	—		1.00	—	
Yes	0.92	(0.88, 0.97)	**<0.001**	0.93	(0.88, 0.98)	**<0.01**
**Sex of household head**						
Female	1.00	—		1.00	—	
Male	1.01	(0.94, 1.08)	0.86	1.07	(0.99, 1.15)	<**0.10**
**Age of household head**						
Less than 60 years	1.00	—		1.00	—	
Over 60 years	1.40	(1.30, 1.51)	**<0.001**	1.43	(1.33, 1.55)	**<0.001**
**(Intercept)**	0.20	(0.17, 0.23)	**<0.001**	0.18	(0.14, 0.24)	**<0.001**

OR = Odds Ratio, Adj. OR = Adjusted Odds Ratio, CI = Confidence Interval

The results of the logistic regression are very similar to the ones obtained from the multilevel logistic regression model. There are slight changes in the estimated AOR and CI’s. Table 3 shows that, for the multilevel logistic regression model, after adjusting for other variables, there exists a positive association between presence of old age person in the household and CHE. Households with at least one person over the age 65 years had 50% higher odds of facing CHE compared to households with no person over the age of 65 years. Households with children under the age 15 years had 28% lower odds of experiencing CHE (AOR 0.72 with 95% CI: 0.68, 0.77) compared to households with no children under the age 15. Higher wealth index was seen to be significantly and negatively associated with exposure to CHE. Households in the richest wealth class had 34% lower (AOR 0.66 with 95% CI: 0.61, 0.72) odds of facing CHE compared to the poorest households. Presence of chronic diseases was found to have the strongest association with CHE. Households with at least one member who had an episode of chronic disease in the past year had 5.45 times higher odds of experiencing CHE (AOR 5.45 with 95% CI: 5.14, 5.77) compared to households who did not have any episode of chronic disease. Place of residence and division were also found to be significantly associated with CHE where, odds of CHE were the highest in Barisal and rural regions and the lowest in Mymensingh, Dhaka and city corporation areas. Among the households where the household head has received education, the odds of incurred CHE are 7% lower compared to the households where the head did not receive any education. Households where the head is male were at a 7% higher risk of CHE compared to the female lead households. This variable was found to be insignificant in the logistic regression model but significant at 10% level of significance in the multilevel logistic regression model. Age of the household head is also a significant predictor of CHE. Households with head aged above 60 years had 43% higher odds of facing CHE compared to households where the head is aged below 60 years.

The interclass correlation (ICC) for the model corresponding to Table 3 was calculated as 19.65%. Thus, almost 20% of the total variation in the chance of CHE incurred by households is explained by the difference between the clusters (PSUs). Although we did not come across any explicit threshold for the ICC to justify the application of multilevel modeling, several studies in the literature have effectively utilized multilevel modeling even with ICC values considerably below 20% [39]. The standard deviation of the random intercepts was found to be 0.7224.

Table 4 shows the results obtained from the logistic and multilevel logistic regression where the response variable catastrophic health expenditure takes the value 1 when a household’s health expenditures exceed its total expenditure data-driven region-specific thresholds and takes the value 0 otherwise.

**Table 4:** Logistic and multilevel logistic regression model results with total expenditure data-driven region-specific threshold.

Variables	Logistic			Multilevel Logistic		
	Adj. OR	95% CI	p-value	Adj. OR	95% CI	p-value
**Presence of old age person (65+)**						
No	1.00	—		1.00	—	
Yes	1.47	(1.37, 1.58)	**<0.001**	1.48	(1.38, 1.59)	**<0.001**
**Presence of child (< 15)**						
No	1.00	—		1.00	—	
Yes	0.73	(0.70, 0.77)	**<0.001**	0.72	(0.68, 0.76)	**<0.001**
**Wealth Index**						
Poorest	1.00	—		1.00	—	
Second	0.93	(0.86, 1.00)	**0.040**	0.93	(0.86, 1.00)	**0.059**
Third	0.99	(0.92, 1.07)	0.86	0.98	(0.90, 1.06)	0.58
Fourth	0.96	(0.89, 1.04)	0.30	0.95	(0.88, 1.03)	0.19
Richest	0.96	(0.89, 1.04)	0.29	0.94	(0.86, 1.02)	0.15
**Presence of chronic disease**						
No	1.00	—		1.00	—	
Yes	5.32	(5.06, 5.59)	**<0.001**	5.20	(4.92, 5.50)	**<0.001**
**Place of residence**						
City Corporation	1.00	—		1.00	—	
Rural	1.33	(1.18, 1.50)	**<0.001**	1.34	(1.11, 1.61)	**0.003**
Urban	1.23	(1.09, 1.39)	**<0.001**	1.23	(1.01, 1.50)	**0.040**
**Division**						
Barisal	1.00	—		1.00	—	
Chittagong	0.57	(0.52, 0.62)	**<0.001**	0.54	(0.46, 0.62)	**<0.001**
Dhaka	0.42	(0.38, 0.45)	**<0.001**	0.40	(0.35, 0.47)	**<0.001**
Khulna	0.45	(0.41, 0.49)	**<0.001**	0.44	(0.38, 0.51)	**<0.001**
Mymensingh	0.37	(0.33, 0.42)	**<0.001**	0.36	(0.30, 0.44)	**<0.001**
Rajshahi	0.44	(0.40, 0.48)	**<0.001**	0.43	(0.37, 0.51)	**<0.001**
Rangpur	0.44	(0.40, 0.48)	**<0.001**	0.42	(0.36, 0.50)	**<0.001**
Sylhet	0.41	(0.37, 0.46)	**<0.001**	0.40	(0.33, 0.49)	**<0.001**
**Head received education**						
No	1.00	—		1.00	—	
Yes	1.02	(0.97, 1.07)	0.43	1.03	(0.98, 1.09)	0.24
**Sex of household head**						
Female	1.00	—		1.00	—	
Male	0.93	(0.86, 0.99)	**0.024**	0.98	(0.91, 1.05).	0.55
**Age of household head**						
Less than 60 years	1.00	—		1.00	—	
Over 60 years	1.41	(1.31, 1.51)	**<0.001**	1.44	(1.33, 1.55)	**<0.001**
**(Intercept)**	0.24	(0.20, 0.28)	**<0.001**	0.23	(0.18, 0.29)	**<0.001**

OR = Odds Ratio, Adj. OR = Adjusted Odds Ratio, CI = Confidence Interval

The results of this model are fairly similar to the nonfood expenditure-based threshold. Here, in the multilevel logistic regression model, the odds of incurring CHE is 48% higher in households where there is at least one person above the age 65 years where the reference group is the households with no person above the age 65 years, assuming that the other variables are held fixed. Place of residence, division, presence of children are also found to be significantly associated with CHE. Wealth index was also found to have significant association with CHE. The association between presence of chronic disease and CHE was very high in this case as well. The odds of households with at least one episode of chronic disease in the past one year experiencing CHE was seen to be 5.2 times more compared to households that did not have any episode of CHE during the same time. The association between CHE and household heads education and sex are found insignificant in this model.

The ICC for this model was found 18.99%. So, when the total expenses threshold is used, almost 19% of the total variation in the chance of CHE incurring in households is explained by the difference between the clusters (PSUs). The standard deviation of the random intercepts was found to be 0.7051.

A likelihood ratio test was performed to test between the logistic regression model and the multilevel logistic regression model. When the response variable was CHE using the nonfood threshold, the likelihood ratio test had the p-value: <0.001. Thus, we may conclude that, the multilevel logistic regression model is more appropriate compared to the logistic regression model.

The Generalized Variance Inflation Factors (GVIF) were evaluated in order to test multicollinearity in the models. Tables 5 and 6 shows the calculated squared GVIF for two models, one with response variable as CHE when its defined using the data-driven region-specific nonfood expenditure threshold and the total expenditure threshold, respectively. For both models, the squared GVIF’s are less than 5 which indicate that there is no multicollinearity in both of the models.

**Table 5:** GVIF for multilevel logistic regression model for CHE with nonfood expenditure data-driven region-specific threshold.

Variables	GVIF	df	Adjusted GVIF	Squared Adjusted GVIF
Presence of old age person (65+)	1.38	1	1.17	1.38
Presence of child (< 15)	1.16	1	1.08	1.16
Wealth Index	1.12	4	1.01	1.03
Presence of chronic disease	1.03	1	1.02	1.03
Place of residence	1.08	2	1.02	1.04
Division	1.11	7	1.01	1.02
Head received education	1.10	1	1.05	1.10
Sex of household head	1.04	1	1.02	1.04
Age of household head	1.50	1	1.23	1.50

df = degrees of freedom

**Table 6:** GVIF for multilevel logistic regression model for CHE with total expenditure data-driven region-specific threshold.

Variables	GVIF	df	Adjusted GVIF	Squared Adjusted GVIF
Presence of old age person(65+)	1.37	1	1.17	1.37
Presence of child (< 15)	1.15	1	1.07	1.15
Wealth Index	1.12	4	1.01	1.03
Presence of chronic disease	1.03	1	1.02	1.03
Place of residence	1.09	2	1.02	1.04
Division	1.11	7	1.01	1.02
Head received education	1.10	1	1.05	1.10
Sex of household head	1.04	1	1.02	1.04
Age of household head	1.49	1	1.22	1.49

df = degrees of freedom

### Sensitivity analysis

**Fig 8:**
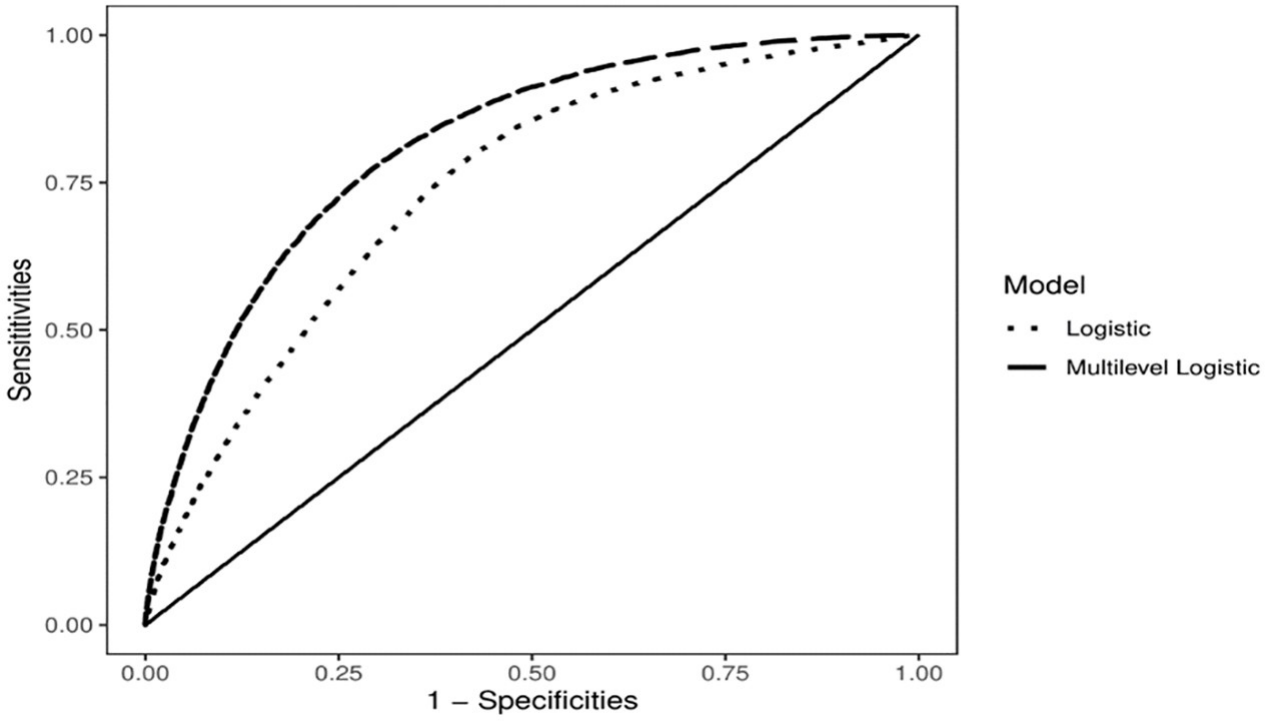
ROC curve for logistic and multilevel logistic regression models with total expenditure data-driven region-specific thresholds (Author’s own compilation).

Fig 8 shows the ROC curve for both multiple logistic regression model and the multilevel logistic regression model to compare the classification performance of the two models at all classification thresholds. The area under the ROC curve corresponding to the multiple logistic regression model is: 0.7382. When considering the multilevel logistic regression, this area increases to 0.8133. Thus, the multilevel logistic regression model has better discriminating ability.

**Fig 9:**
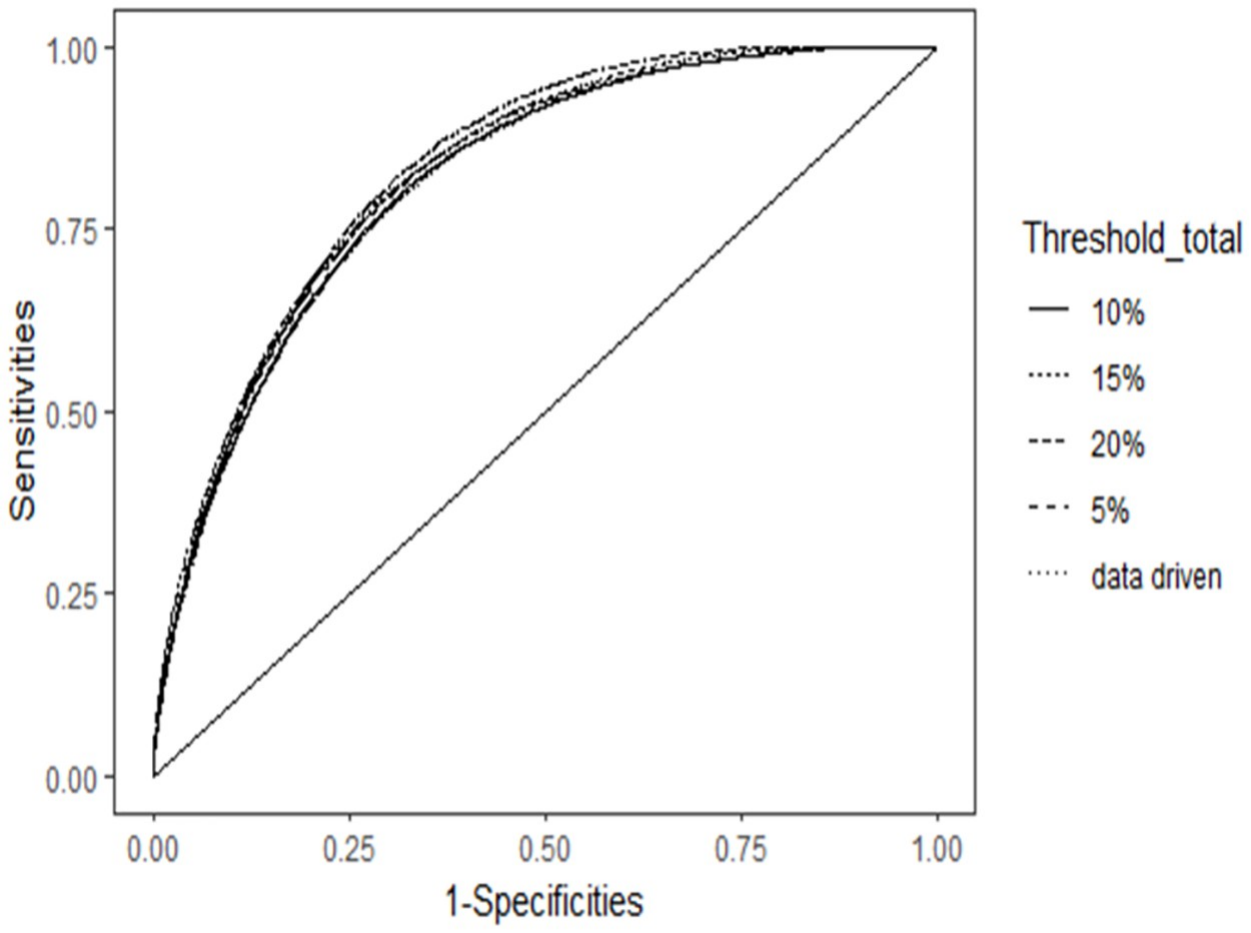
ROC curve for different total expenditure thresholds of CHE (Source: Author’s own compilation).

**Fig 10:**
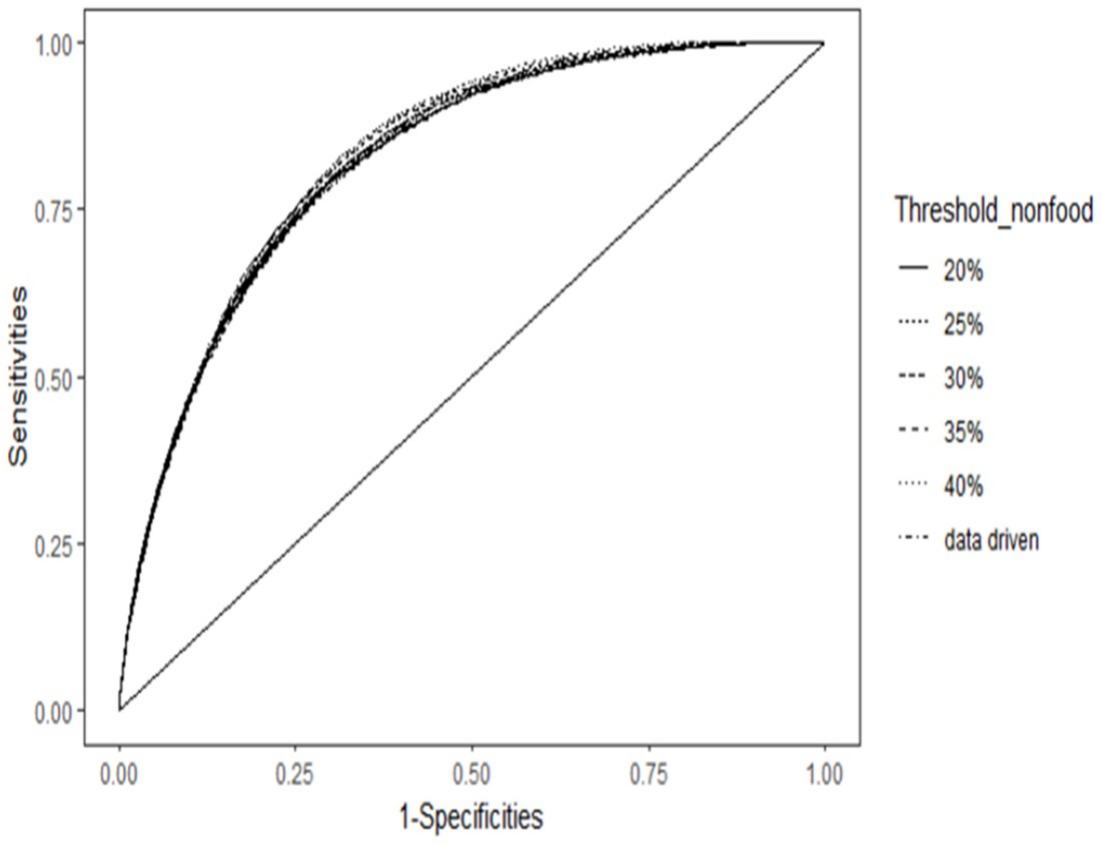
ROC curve for different nonfood expenditure thresholds of CHE (Source: Author’s own compilation).

A key target of this study is to determine the appropriate threshold to define CHE. By conducting a sensitivity analysis of various thresholds using ROC curve in Fig 9, it has been observed that the predictive performance of the models remains relatively unaffected by the choice of the WHO and data-driven region-specific thresholds. A similar pattern is observed when examining the nonfood expenditure threshold. Fig 10 illustrates the ROC curve for various thresholds of nonfood expenditure. It is depicted that the data-driven region-specific threshold has quite similar area under the curve compare to the WHO threshold.

## Conclusion

The primary goal of this study is to identify the districts of Bangladesh that have a heightened risk of CHE and to showcase the discrepancies in the prevalence of CHE across various districts. Another objective is to explore whether the threshold used to define CHE differs across regions. Once a suitable threshold is identified, a multilevel logistic regression model is utilized to identify the significant factors that contribute to CHE.

The district-wise analysis indicates a significant variation in the prevalence of CHE, particularly when a 10% threshold of total expenditure is considered. Among the districts studied, several eastern and southern regions such as, Comilla, Jhalokati, Barguna, Noakhali, Barisal, Bhola, Cox’s Bazar, Rajbari, Thakurgaon, and Pirojpur exhibits the highest risk of CHE. The high frequency of chronic diseases in those districts, coupled with their economic situations, could offer a potential rationale for these results. The results are in line with Begum and Hamid, 2021, as districts with a high incidence of catastrophic health expenditure are also found to be situated near rivers [14]. These findings are consistent with previous research indicating that the prevalence of CHE varies geographically [28]. Furthermore, this study observes that the thresholds for defining CHE varies widely across regions, which is in agreement with earlier multi-country analyses [25, 26]. These results suggest that the WHO-recommended 40% nonfood expenditure criterion differs to adequately account for the impoverishment impact of CHE. However, when total expenditures are considered, the region- specific thresholds align more closely with the commonly employed 10% threshold. Analysis shows that, on average, households in Bangladesh allocate 55% of their total expenditures on food and 45% on non-food expenses. Consequently, a lower proportion of non-food expenditures on health expenses are sufficient to push a household below the poverty line. The mapping of CHE based on the data-driven region-specific threshold reveals a robust tool for poverty mapping. Another interesting pattern emerges when mapping the prevalence of chronic diseases and the proportion of food expenditure, indicating a potential association between CHE and chronic disease and the wealth index. This association is justified through the multilevel logistic regression model considering the fixed and random effects. Although the maps of CHE vary considerably from the poverty map of Bangladesh, suggesting that CHE is not yet being considered as a sole factor driving households into poverty, rather the policy makers may emphasize on the CHE issue immediately.

The multilevel logistic regression on nonfood threshold reveals that the presence of chronic disease (AOR 5.45 with 95% CI: 5.14, 5.77) is the most influential variable for CHE, it means that the households with chronic disease have about 5 times higher chance of being poor due to catastrophic heath expenditure compare to the non-chronic diseased households. Other variables, such as the wealth index, the presence of an elderly person in the household (AOR 1.48 with 95% CI: 1.38, 1.59), and the age of the household head (AOR 1.44 with 95% CI: 1.33, 1.55), are also highly significant predictors of CHE. These findings align with those of recent studies [13, 29]. Surprisingly, the presence of children in the household are negatively associated with CHE, which contradicts our initial expectations. However, previous studies also report similar results [13]. Finally, the ROC curve analysis demonstrates that the multilevel logistic regression model outperforms the logistic regression model in terms of its ability to discriminate between households with and without CHE. Our findings align with numerous studies in the literature that consistently demonstrate the superior predictive performance of multilevel modeling when compared to nonhierarchical models [40].

By conducting a comprehensive analysis of the underlying factors contributing to the high prevalence of CHE in Bangladesh, this study has the potential to establish a foundation for policymakers. It sheds light on the districts that require urgent attention to combat CHE, which aligns with the broader goal of achieving the SDGs. Additionally, this research advocates for interventions that aim to decrease the prevalence of chronic diseases in regions with high levels of CHE. It also identifies various socioeconomic and demographic factors that aggravate CHE in these areas. Moreover, the study emphasizes the importance of selecting an appropriate CHE threshold that captures the impoverishing effect of healthcare expenses. The suggested threshold can be used for future investigations on the correlation between CHE and poverty.

The financial strain caused by CHE can lead households into poverty and impede their access to essential healthcare services. By establishing a suitable threshold, a district-level analysis is conducted, revealing notable disparities in the prevalence of CHE. The study’s findings suggest that prioritizing the management of chronic diseases could serve as a critical strategy to improve the health outcomes in Bangladesh. Policymakers may be encouraged to prioritize interventions aimed at reducing the prevalence of chronic diseases in high-risk districts of the country and counting CHE as a core component of poverty mapping. These interventions have the potential to significantly enhance the health outcomes and financial well-being of individuals and communities across the country.

## Supporting information

S1 File (PDF)

S1 Data (ZIP)
