# Supplementary material for: From the local disparities to national realities: Mapping and multilevel modeling of catastrophic health expenditure in Bangladesh using HIES 2016
Source: PLoS One. 2024 Jan 2;19(1):e0290746. doi: 10.1371/journal.pone.0290746 (PMC10760898; doi:10.1371/journal.pone.0290746)
Supplement: S1 File — (PDF) [file pone.0290746.s001.pdf]

```
load("working_data2.R")

library(tidyverse)
library(gtsummary)
library(haven)
library(kableExtra)
library(sf)
library(ggplot2)
library(gridExtra)
library(kableExtra)
library(lme4)
library(car)
library(broom.mixed)
library(pROC)


table_data <- working_data %>%
  mutate(CHE_nonfood = ifelse(hlth_exp_3A >= 0.40*nfexp2,
                              1,0),
         CHE_nonfood_25 = ifelse(hlth_exp_3A >= 0.25*nfexp2,
                                 1,0),
         CHE_total = ifelse(hlth_exp_3A >= 0.1*consexp,
                            1,0),
         head_edu = as.numeric(Head_recieved_edu),
         head_sex = as.numeric(head_sex),
         id_01_name = tolower(id_01_name)
  )

firstup <- function(x) {
```

```
30      substr(x, 1, 1) <- toupper(substr(x, 1, 1))
31      x
32    }
33
34    table_data <- table_data %>%
35      select(CHE_nonfood_25,
36             CHE_total,
37             person_over65,
38             child_under15,
39             wealth.Index,
40             chronic,
41             head_edu,
42             ruc,
43             Division = id_01_name,
44             head_sex,
45             head_age,
46             ) %>%
47      mutate(Division = firstup(Division))
48
49    table_data$CHE_nonfood_25[table_data$CHE_nonfood_25==0] <- "No"
50    table_data$CHE_nonfood_25[table_data$CHE_nonfood_25==1] <- "Yes"
51    table_data$CHE_total[table_data$CHE_total==0] <- "No"
52    table_data$CHE_total[table_data$CHE_total==1] <- "Yes"
53    table_data$person_over65[table_data$person_over65==0] <- "No"
54    table_data$person_over65[table_data$person_over65==1] <- "Yes"
55    table_data$child_under15[table_data$child_under15==0] <- "No"
56    table_data$child_under15[table_data$child_under15==1] <- "Yes"
57    table_data$chronic[table_data$chronic==0] <- "No"
58    table_data$chronic[table_data$chronic==1] <- "Yes"
```

```
table_data$head_edu[table_data$head_edu==1] <- "Yes"
table_data$head_edu[table_data$head_edu==2] <- "No"
table_data$head_sex[table_data$head_sex==1] <- "Male"
table_data$head_sex[table_data$head_sex==2] <- "Female"
table_data$head_age[table_data$head_age==0] <- "Under 60 years"
table_data$head_age[table_data$head_age==1] <- "60+"
table_data$ruc[table_data$ruc==1] <- "Rural"
table_data$ruc[table_data$ruc==2] <- "Urban"
table_data$ruc[table_data$ruc==3] <- "City Corporation"

univariate_table <- tbl_summary(table_data,missing = "no",
                        type = list(c(CHE_nonfood_25,
                                CHE_total,
                                person_over65,
                                child_under15,
                                wealth.Index,
                                chronic,
                                head_edu,
                                head_sex,
                                ruc,
                                Division,
                                head_age)~ "categorical"),
                        statistic = list(
                          all_categorical() ~ "{n}({p})"),
                        label = list(CHE_nonfood_25 ~ "CHE (25% nonfood expenditure)",
                                CHE_total ~ "CHE (10% total expenditure)",
                                person_over65 ~ "Presence of old age (65+)",
                                child_under15 ~ "Presence of child (< 15)",
                                wealth.Index ~ "Wealth Index",
```

```
                                chronic ~ "Presence of chronic disease",
                                head_edu ~ "Head recieved education",
                                head_sex ~ "Sex of household head",
                                ruc ~ "Place of residence",
                                head_age ~ "Age of household head"
                            ))


save(univariate_table,file = "univariate_table.Rdata")

bivariate_table1 <- table_data %>%
  select(-CHE_total) %>%
  tbl_summary(by = CHE_nonfood_25,missing = "no",percent = "row",
              digits = everything() ~ 1,
              type = list(c(person_over65,
                            child_under15,
                            wealth.Index,
                            chronic,
                            head_edu,
                            head_sex,
                            head_age)~ "categorical"),
              statistic = list(
                all_categorical() ~ "{p}%"),
              label = list(person_over65 ~ "Presence of old age (65+)",
                           child_under15 ~ "Presence of child (< 15)",
                           wealth.Index ~ "Wealth Index",
                           chronic ~ "Presence of chronic disease",
                           head_edu ~ "Head recieved education",
                           ruc ~ "Place of residence",
```

```
                head_sex ~ "Sex of household head",
                head_age ~ "Age of household head"
        )) %>%
  modify_column_hide(columns = stat_1) %>%
  add_p()

bivariate_table2 <- table_data %>%
  select(-CHE_nonfood_25) %>%
  tbl_summary(by = CHE_total,missing = "no",digits = everything() ~ 1,
        percent = "row",
        type = list(c(person_over65,
                child_under15,
                wealth.Index,
                chronic,
                head_edu,
                head_sex,
                head_age)~ "categorical"),
        statistic = list(
          all_categorical() ~ "{p}%"),
        label = list(person_over65 ~ "Presence of old age (65+)",
                child_under15 ~ "Presence of child (< 15)",
                wealth.Index ~ "Wealth Index",
                ruc ~ "Place of residence",
                chronic ~ "Presence of chronic disease",
                head_edu ~ "Head recieved education",
                head_sex ~ "Sex of household head",
                head_age ~ "Age of household head"
        )) %>%
  modify_column_hide(columns = stat_1) %>%
```

```
  add_p()


bivariate_table <-
  tbl_merge(
    tbls = list(bivariate_table1, bivariate_table2),
    tab_spanner = c("**CHE (25% nonfood)**", "**CHE (10% total)**")
  )

save(bivariate_table,file = "bivariate_table.Rdata")

working_data$NAME_2[working_data$NAME_2=="CHAPAI NABABGANJ"] <- "NAWABGANJ"
divdat <- working_data %>%
  group_by(NAME_2) %>%
  summarise(propche_nonfood = mean(CHE_nonfood,na.rm=T),
            propche_total = mean(CHE_total,na.rm=T),
            prop_chronic = mean(chronic,na.rm = T))

mapdat <- read_sf("E:/Project/BD
MAP/bgd_adm_bbs_20201113_SHP/bgd_admbnda_adm2_bbs_20201113.shp")
mapdat$CHE_nonfood <- divdat$propche_nonfood
mapdat$CHE_total <- divdat$propche_total
mapdat$prop_chronic <- divdat$prop_chronic

map_chonic <- ggplot(mapdat)+
  geom_sf(aes(fill=prop_chronic))+
  scale_fill_gradient2(
    low = "white",
    high = "red4",
```

```
    mid = "orangered",
    midpoint = 0.6,
  )+theme_bw() +labs(x = NULL, y = NULL, fill = "Chronic") +
  theme(axis.title.x = element_blank(),
        axis.title.y = element_blank(),
        axis.line = element_blank(),
        axis.text = element_blank(),
        axis.ticks = element_blank()) +
  geom_sf_text(aes(label = ADM2_EN), colour = "black",size=2.6

save(map_chonic, file = "map_ch.Rdata")

map_nonfood <- ggplot(mapdat)+
  geom_sf(aes(fill=CHE_nonfood))+
  scale_fill_gradient2(
    low = "white",
    high = "red4",
    mid = "orangered",
    midpoint = 0.3,
  )+theme_bw() +labs(x = NULL, y = NULL, fill = "CHE") +
  theme(axis.title.x = element_blank(),
        axis.title.y = element_blank(),
        axis.line = element_blank(),
        axis.text = element_blank(),
        axis.ticks = element_blank()) +
  geom_sf_text(aes(label = ADM2_EN), colour = "black",size=2.6)
save(map_nonfood,file = "map_nonfood.Rdata")

map_total <- ggplot(mapdat)+
```

```
204     geom_sf(aes(fill=CHE_total))+
205     scale_fill_gradient2(
206       low = "white",
207       high = "red4",
208       mid = "orangered",
209       midpoint = 0.3,
210     )+
211     theme_bw() +labs(x = NULL, y = NULL, fill = "CHE") +
212     theme(axis.title.x = element_blank(),
213           axis.title.y = element_blank(),
214           axis.line = element_blank(),
215           axis.text = element_blank(),
216           axis.ticks = element_blank()) +
217     geom_sf_text(aes(label = ADM2_EN), colour = "black",size=2.6)
218
219   save(map_total,file = "map_total.Rdata")
220
221
222
223   poverty_data <- read_dta("E:\\Project\\HIES2016\\poverty_indicators2016.dta")
224   poverty_data <- select(poverty_data, hhold, zu16, zl16,consexp2, member , pcexp ) %>%
225     mutate(pov_line=(zu16+zl16)/2)
226
227   dat12 <- merge(working_data,poverty_data,by="hhold") %>%
228     mutate(pov_before=ifelse(pcexp<pov_line,1,0)) %>%
229     mutate(reduced_exp = consexp2.y - hlth_exp_9d2) %>%
230     mutate(preduced_exp=reduced_exp/member) %>%
231     mutate(pov_ind_after_reduc=ifelse(preduced_exp<pov_line,1,0)) %>%
232     mutate(new_hh_after_red=case_when(
```

```
                pov_before==0 & pov_ind_after_reduc==1 ~1,
                pov_before==1 & pov_ind_after_reduc==1 ~0,
                pov_before==0 & pov_ind_after_reduc==0 ~0,
                pov_before==1 & pov_ind_after_reduc==0 ~0,
                TRUE ~ NA_real_
              ))

data_driven_threshold1 <- filter(dat12,new_hh_after_red==1) %>%
  mutate(percent_of_nonfood=(hlth_exp_9d2/nfexp2)*100) %>%
  group_by(stratum16) %>%
  summarise(Threshold_nfood=mean(percent_of_nonfood))


data_driven_threshold2 <- filter(dat12,new_hh_after_red==1) %>%
  mutate(percent_of_totalexp=(hlth_exp_9d2/consexp2.x)*100) %>%
  group_by(stratum16) %>%
  summarise(Threshold_totalexp=mean(percent_of_totalexp))

d_d_threshold <- merge(data_driven_threshold1,data_driven_threshold2,
                       by="stratum16")

d_d_threshold <- d_d_threshold %>% mutate(stratum16=as.character(stratum16))
d_d_threshold$stratum16[d_d_threshold$stratum16=="1"] <- "Barisal (Rural)"
d_d_threshold$stratum16[d_d_threshold$stratum16=="2"] <- "Barisal (Urban)"
d_d_threshold$stratum16[d_d_threshold$stratum16=="3"] <- "Chittagong (Rural)"
d_d_threshold$stratum16[d_d_threshold$stratum16=="4"] <- "Chittagong (Urban)"
d_d_threshold$stratum16[d_d_threshold$stratum16=="5"] <- "Chittagong (City Corp.)"
d_d_threshold$stratum16[d_d_threshold$stratum16=="6"] <- "Dhaka (Rural)"
d_d_threshold$stratum16[d_d_threshold$stratum16=="7"] <- "Dhaka (Urban)"
```

```
d_d_threshold$stratum16[d_d_threshold$stratum16=="8"] <- "Dhaka (City Corp.)"
d_d_threshold$stratum16[d_d_threshold$stratum16=="9"] <- "Khulna (Rural)"
d_d_threshold$stratum16[d_d_threshold$stratum16=="10"] <- "Khulna (Urban)"
d_d_threshold$stratum16[d_d_threshold$stratum16=="11"] <- "Khulna (City Corp.)"
d_d_threshold$stratum16[d_d_threshold$stratum16=="12"] <- "Rajshahi (Rural)"
d_d_threshold$stratum16[d_d_threshold$stratum16=="13"] <- "Rajshahi (Urban)"
d_d_threshold$stratum16[d_d_threshold$stratum16=="14"] <- "Rajshahi (City Corp.)"
d_d_threshold$stratum16[d_d_threshold$stratum16=="15"] <- "Sylhet (Rural)"
d_d_threshold$stratum16[d_d_threshold$stratum16=="16"] <- "Sylhet (Urban)"
names(d_d_threshold) <- c("Region", "Threshold (%nonfood)", "Threshold (%total)")


save(d_d_threshold, file="d_d_threshold.Rdata")

dat2map <- merge(working_data,data_driven_threshold1) %>%
   mutate(CHE_dd_nonf = ifelse(hlth_exp_3A >= Threshold_nfood*nfexp2/100, 1,0)) %>%
  group_by(NAME_2) %>%
  summarise(CHE_dd_nonf=mean(CHE_dd_nonf,na.rm=T))
mapdat$CHE_dd_nonfood <- dat2map$CHE_dd_nonf

map_dd_nonf <- ggplot(mapdat)+
  geom_sf(aes(fill=CHE_dd_nonfood))+
  scale_fill_gradient2(
    low = "white",
    high = "red4",
    mid = "orangered",
    midpoint = 0.3,
  )+
  theme_bw() +labs(x = NULL, y = NULL, fill = "CHE") +
```

```
291     theme(axis.title.x = element_blank(),
292           axis.title.y = element_blank(),
293           axis.line = element_blank(),
294           axis.text = element_blank(),
295           axis.ticks = element_blank()) +
296     geom_sf_text(aes(label = ADM2_EN), colour = "black",size=2.6)
297
298   save(map_dd_nonf,file="map_dd_nonf.Rdata")
299
300
301   dat3map <- merge(working_data,data_driven_threshold2) %>%
302     mutate(CHE_dd_total = ifelse(hlth_exp_3A >= Threshold_totalexp*consexp/100, 1,0)) %>%
303     group_by(NAME_2) %>%
304     summarise(CHE_dd_total=mean(CHE_dd_total,na.rm=T))
305   mapdat$CHE_dd_total <- dat3map$CHE_dd_total
306
307   map_dd_total <- ggplot(mapdat)+
308     geom_sf(aes(fill=CHE_dd_total))+
309     scale_fill_gradient2(
310       low = "white",
311       high = "red4",
312       mid = "orangered",
313       midpoint = 0.3,
314     )+
315     theme_bw() +labs(x = NULL, y = NULL, fill = "CHE") +
316     theme(axis.title.x = element_blank(),
317           axis.title.y = element_blank(),
318           axis.line = element_blank(),
319           axis.text = element_blank(),
```

```
320         axis.ticks = element_blank()) +
321   geom_sf_text(aes(label = ADM2_EN), colour = "black",size=2.6)
322
323  save(map_dd_total,file="map_dd_total.Rdata")
324
325  prop_nonfood <- working_data %>%
326   mutate(prop_nonfood = nfexp2/consexp) %>%
327   group_by(NAME_2) %>%
328   summarise(prop_nonfood = mean(prop_nonfood, na.rm = T))
329
330  mapdat$Proportion_nonfood <- prop_nonfood$prop_nonfood
331
332  map_prop_nonfood <- ggplot(mapdat)+
333   geom_sf(aes(fill=Proportion_nonfood))+
334   scale_fill_gradient2(
335    low = "white",
336    high = "red4",
337    mid = "orangered",
338    midpoint = median(mapdat$Proportion_nonfood),
339   )+
340   theme_bw() +labs(x = NULL, y = NULL, fill = "Prop. Nonfood") +
341   theme(axis.title.x = element_blank(),
342         axis.title.y = element_blank(),
343         axis.line = element_blank(),
344         axis.text = element_blank(),
345         axis.ticks = element_blank()) +
346   geom_sf_text(aes(label = ADM2_EN), colour = "black",size=2.6)
347  save(map_prop_nonfood,file="map_prop_nonfood.Rdata")
348
```

```
prop_food <- working_data %>%
  mutate(prop_food = fexp/consexp) %>%
  group_by(NAME_2) %>%
  summarise(prop_food = mean(prop_food, na.rm = T))

mapdat$Proportion_food <- prop_food$prop_food

map_prop_food <- ggplot(mapdat)+
  geom_sf(aes(fill=Proportion_food))+
  scale_fill_gradient2(
    low = "white",
    high = "red4",
    mid = "orangered",
    midpoint = median(mapdat$Proportion_food),
  )+
  theme_bw() +labs(x = NULL, y = NULL, fill = "Prop. Food") +
  theme(axis.title.x = element_blank(),
        axis.title.y = element_blank(),
        axis.line = element_blank(),
        axis.text = element_blank(),
        axis.ticks = element_blank()) +
  geom_sf_text(aes(label = ADM2_EN), colour = "black",size=2.6)

save(map_prop_food,file="map_prop_food.Rdata")

save(mapdat,file="mapdat.Rdata")
model_data <- working_data %>%
  mutate(
      CHE_nonfood = ifelse(hlth_exp_3A >= 0.25*nfexp2,
```

```
                              1,0),
        CHE_total = ifelse(hlth_exp_3A >= 0.1*consexp,
                           1,0),
        Division = tolower(id_01_name)
    )

firstup <- function(x) {
  substr(x, 1, 1) <- toupper(substr(x, 1, 1))
  x
}

model_data <- model_data %>%
  mutate(prop_foodexp = fexp/consexp,
         prop_nonfoodexp = nfexp2/consexp,
         Division = firstup(Division)
         )

put_vector_in_parenthesis <- function(values){
  put_vector_in_parenthesis <- function(value){
    if(is.na(value)){
      return(value)
    }
    else{
      return(paste0("(",value,")"))
    }
  }
  new_values <- sapply(values, put_vector_in_parenthesis)
  return(new_values)
}
```

```
407
408     model_data$CHE_nonfood[model_data$CHE_nonfood==0] <- "No"
409     model_data$CHE_nonfood[model_data$CHE_nonfood==1] <- "Yes"
410     model_data$CHE_total[model_data$CHE_total==0] <- "No"
411     model_data$CHE_total[model_data$CHE_total==1] <- "Yes"
412     model_data$person_over65[model_data$person_over65==0] <- "No"
413     model_data$person_over65[model_data$person_over65==1] <- "Yes"
414     model_data$child_under15[model_data$child_under15==0] <- "No"
415     model_data$child_under15[model_data$child_under15==1] <- "Yes"
416     model_data$chronic[model_data$chronic==0] <- "No"
417     model_data$chronic[model_data$chronic==1] <- "Yes"
418     model_data$head_edu[model_data$head_edu==1] <- "Yes"
419     model_data$head_edu[model_data$head_edu==2] <- "No"
420     model_data$head_sex[model_data$head_sex==1] <- "Male"
421     model_data$head_sex[model_data$head_sex==2] <- "Female"
422     model_data$head_age[model_data$head_age==0] <- "Less than 60 years"
423     model_data$head_age[model_data$head_age==1] <- "Over 60 years"
424     model_data$Head_recieved_edu <- as.numeric(model_data$Head_recieved_edu)
425     model_data$Head_recieved_edu[model_data$Head_recieved_edu == 2] <- "No"
426     model_data$Head_recieved_edu[model_data$Head_recieved_edu == 1] <- "Yes"
427     model_data$ruc[model_data$ruc==1] <- "Rural"
428     model_data$ruc[model_data$ruc==2] <- "Urban"
429     model_data$ruc[model_data$ruc==3] <- "City Corporation"
430
431     M0 <- glmer(factor(CHE_nonfood) ~ ( 1 | psu), data=model_data, family = "binomial")
432     icc <- M0@theta[1]^2/ (M0@theta[1]^2 + (3.14159^2/3))
433     icc
434     final_mod2<- glmer(factor(CHE_nonfood)~as.factor(person_over65)+
435                        factor(child_under15)+
```

```
                  factor(wealth.Index)+
                  factor(chronic)+
                  factor(ruc)+
                  factor(Division)+
                  factor(Head_recieved_edu)+
                  factor(head_sex)+
                  factor(head_age)+
                  (1|psu),
                data = model_data,
                family = "binomial",nAGQ = 0
               )
model_table1 <- final_mod2 %>% tbl_regression(exponentiate = TRUE,label = list(
  `as.factor(person_over65)` ~ "Presence of old age person (65+)",
  `factor(child_under15)` ~ "Presence of child (< 15) ",
  `factor(wealth.Index)` ~ "Wealth Index",
  `factor(chronic)` ~ "Presence of chronic disease",
  `factor(Head_recieved_edu)` ~ "Head recieved education",
  `factor(head_sex)` ~ "Sex of household head",
  `factor(head_age)` ~ "Age of household head",
  `factor(ruc)` ~ "Place of residence",
  `factor(Division)` ~ "Division"
),
                    pvalue_fun = ~ style_pvalue(.x, digits = 2),
                    add_estimate_to_reference_rows = TRUE,
                    intercept = TRUE) %>%
  modify_table_body(~ .x %>%
                mutate(ci = put_vector_in_parenthesis(ci))) %>%
  modify_header(update = list("label" = "**Variables**",
                    "estimate" = "**Adj. OR**"))
```

```
save(model_table1,file="model_table1.Rdata")

final_mod3<- glmer(factor(CHE_total)~as.factor(person_over65)+
                     factor(child_under15)+
                     factor(wealth.Index)+
                     factor(chronic)+
                     factor(ruc)+
                     factor(Division)+
                     factor(Head_recieved_edu)+
                     factor(head_sex)+
                     factor(head_age)+
                     (1|psu),
                   data = model_data,
                   family = "binomial",nAGQ = 0
)

model_table2 <- final_mod3 %>% tbl_regression(exponentiate = TRUE,label = list(
  `as.factor(person_over65)` ~ "Presence of old age person (65+)",
  `factor(child_under15)` ~ "Presence of child (< 15) ",
  `factor(wealth.Index)` ~ "Wealth Index",
  `factor(chronic)` ~ "Presence of chronic disease",
  `factor(Head_recieved_edu)` ~ "Head recieved education",
  `factor(head_sex)` ~ "Sex of household head",
  `factor(head_age)` ~ "Age of household head",
  `factor(ruc)` ~ "Place of residence",
  `factor(Division)` ~ "Division"
),
pvalue_fun = ~ style_pvalue(.x, digits = 2),
```

```
494   add_estimate_to_reference_rows = TRUE,
495   intercept = TRUE) %>%
496    modify_table_body(~ .x %>%
497                        mutate(ci = put_vector_in_parenthesis(ci))) %>%
498    modify_header(update = list("label" = "**Variables**",
499                                "estimate" = "**Adj. OR**"))
500
501   M0 <- glmer(factor(CHE_total) ~ ( 1 | psu), data=model_data, family = "binomial")
502   icc <- M0@theta[1]^2/ (M0@theta[1]^2 + (3.14159^2/3))
503   icc
504   save(model_table2,file="model_table2.Rdata")
505   model_logis <- glm(factor(CHE_nonfood)~
506                        as.factor(person_over65)+
507                         factor(child_under15)+
508                         factor(wealth.Index)+
509                         factor(chronic)+
510                         factor(ruc)+
511                         factor(Division)+
512                         factor(Head_recieved_edu)+
513                         factor(head_sex)+
514                         factor(head_age),
515                        data = model_data,
516                        family = "binomial"
517   )
518
519   roc(final_mod2@resp$y,fitted(final_mod2),legacy.axes = T) ->model_roc1
520   coords(model_roc1,"best")
521   roc_graph1 <- ggroc(model_roc1, legacy.axes = TRUE, col = "#377eb8", size = 1) +
522    geom_segment(aes(x = 0, xend = 1, y = 0, yend = 1), color="red") +
```

```
  theme_bw() +
  labs(x = "False positive rate",
       y = "True positive rate") +
  theme(panel.grid = element_blank())
save(roc_graph1,file = "roc1.Rdata")
roc(model_logis$y,fitted(model_logis), legacy.axis = T) ->model_roc2
coords(model_roc2,"best")
ggroc(model_roc2, legacy.axes = TRUE, col = "#377eb8", size = 1) +
  geom_segment(aes(x = 0, xend = 1, y = 0, yend = 1), color="red")+
  theme_bw() +
  labs(x = "False positive rate",
       y = "True positive rate") +
  theme(panel.grid = element_blank()) -> roc_graph2
save(roc_graph2,file = "roc2.Rdata")
Sensititivities <- c(model_roc1$sensitivities,model_roc2$sensitivities)
Specificities <- c(model_roc1$specificities,model_roc2$specificities)
Model <- c(rep("Multilevel Logistic",34297),rep("Logistic",3800))
roc_data <- data.frame(Sensititivities,Specificities,Model)
roc_both <- ggplot(data = roc_data)+
  geom_line(mapping = aes(x=1-Specificities , y=Sensititivities, color = Model),linewidth=1)+
  geom_segment(aes(x = 0, xend = 1, y = 0, yend = 1), color="red")+
  theme_bw()+
  theme(panel.grid = element_blank())+
  scale_color_manual(values=c("#00AFBB", "#E7B800"))
save(roc_both,file = "roc_both.Rdata")
save(roc_data,file="roc_data.Rdata")
vif1 <- as_tibble(vif(final_mod2))
vif1$Variables <-  c("Presence of old age person(65+)",
                     "Presence of child (< 15) ",
```

```
                  "Wealth Index",
                  "Presence of chronic disease",
                  "Place of residence",
                  "Division",
                   "Head recieved education",
                  "Sex of household head",
                   "Age of household head"
                  )
 vif1 <-  select(vif1,Variables,GVIF,    Df, Adjusted_GVIF = `GVIF^(1/(2*Df))`) %>%
   mutate(sq_gv = Adjusted_GVIF^2)
 names(vif1)[4] <- "Adjusted GVIF"
 names(vif1)[5] <- "Squared Adjusted GVIF"
vif_tab1 <- kbl(vif1)
vif2 <- as_tibble(vif(final_mod3))
vif2$Variables <-  c("Presence of old age person(65+)",
                   "Presence of child (< 15) ",
                   "Wealth Index",
                   "Presence of chronic disease",
                   "Place of residence",
                   "Division",
                   "Head recieved education",
                   "Sex of household head",
                   "Age of household head"
)
vif2 <-  select(vif2,Variables,GVIF,    Df, Adjusted_GVIF = `GVIF^(1/(2*Df))`) %>%
  mutate(sq_gv = Adjusted_GVIF^2)
names(vif2)[4] <- "Adjusted GVIF"
names(vif2)[5] <- "Squared Adjusted GVIF"
vif_tab2 <- kbl(vif2)
```

```
581 save(vif1,file = "vif1.Rdata")
582 save(vif2,file = "vif2.Rdata")
```
